# A rapid clinical deterioration of a cervical exophytic intradural intramedullary sporadic hemangioblastoma diagnosed during pregnancy

**DOI:** 10.3171/2023.7.FOCVID2354

**Published:** 2023-10-01

**Authors:** Ufuk Erginoglu, Abdullah Keles, Cagdas Ataoglu, Seyit Kağan Başarslan, Mustafa K. Baskaya

**Affiliations:** Department of Neurological Surgery, School of Medicine and Public Health, University of Wisconsin, Madison, Wisconsin

**Keywords:** hemangioblastoma, intradural, intramedullary, pregnancy, trimester, spinal, tumor

## Abstract

Hemangioblastomas are benign CNS tumors that can occur sporadically or in conjunction with von Hippel–Lindau disease. While 2% of spinal cord tumors are hemangioblastomas, combined cervical hemangioblastomas and pregnancy is rare. Some reports suggest that hemodynamic and hormonal changes in pregnancy might increase hemangioblastoma growth and aggravate symptoms. Urgent tumor removal is required when neurological problems deteriorate after failed symptomatic treatment. Neurosurgeons should collaborate with anesthesiologists and obstetricians in such cases. Herein, the authors present the first known video case of a sporadic cervical hemangioblastoma diagnosed during pregnancy that required urgent surgery due to failed symptomatic treatment and progressive clinical deterioration.

## Transcript

This surgical video depicts a cervical exophytic intradural intramedullary sporadic hemangioblastoma. Here, we provided a comprehensive discussion of the treatment options available for pregnant women diagnosed with spinal hemangioblastomas.

### 0:35 Background Information.

Hemangioblastomas are uncommon benign vascular tumors of the spinal cord, accounting for less than 2% of all spinal cord tumors.^1,2^ Although the co-occurrence of hemangioblastoma with pregnancy is infrequent, some studies indicate that alterations in hemodynamics and hormonal status during pregnancy may accelerate tumor growth and exacerbate symptoms.^3–8^

### 0:55 Case Presentation.

In this video, we present a case of a 40-year-old 26 weeks pregnant female who presented to our department with a C2–3 spinal cord tumor at 16 weeks of gestation. The patient’s complaints had begun in the early stages of her pregnancy and included numbness in her hands and weakness in her right arm. Once her complaints persisted, she was referred to us after an MRI indicated a cervical spine mass. The first neurological exam at our clinic revealed that her right arm was slightly weaker than her left arm (4/5 strength). She was then started on dexamethasone, whereupon the weakness of her extremities slightly improved. She was subsequently intensively monitored for weeks by our neurological surgery department and by maternal fetal medicine, obstetrics, anesthesiology, and radiology. However, by 26 weeks of pregnancy, she had experienced progressive weakness. Her left upper-limb strength was 2/5, and left-lower limb strength was 4/5, along with right-sided hemiplegia and respiratory distress.

### 1:57 Preoperative Imaging.

The MRI revealed an expansile, homogeneously enhancing intramedullary mass within the cervical cord at the level of the C2–3 measuring approximately 2.3 × 1.5 × 3.7 cm. The repeat MRI of her spine showed a slight increase in her known spinal mass. Although she was started on dexamethasone, the weakness of her bilateral upper extremities did not improve significantly with steroids.

### 2:23 Management.

Patients with hemangioblastoma of the central nervous system who are pregnant may initially treated with symptomatically. The treatment of these patients is complex and must be customized to their neurological symptoms and gestational age.^2,5,6,8^

In the present case, since the patient’s neurological impairments continued to worsen despite symptomatic treatment, and since she also experienced some respiratory distress, she was transferred to the operating theater for a C-section followed by the excision of the cervical tumor.

### 2:50 Patient Positioning and Skin Incision.

Under general anesthesia, the patient was placed in a prone position, and her head was slightly flexed and fixed with a Mayfield head holder.

The intraoperative positioning must be determined by the gestational age, location, and size of the lesion. Because there is less aortocaval compression by the pregnant uterus throughout the first and early second trimesters, the prone position is recommended. Following 12 weeks of pregnancy, the left lateral posture is recommended to avoid aortocaval compression. The patient was placed in the prone position since our decision was to perform tumor surgery after C-section.^2,5^

Of note, neuromonitoring of SSEPs and MEPs did not deviate from the baselines obtained prior to prone positioning.

### 3:32 Incision.

The incision was made from the lower occiput to C4. Self-retaining retractors were placed. We then removed the spinal processes with a Leksell rongeur and used a high-speed drill to create our laminectomies of C2, C3, and C4. We found the dura was highly swollen and elected to do a C1 laminectomy.

### 3:47 Dural Opening.

After the dural opening, we immediately noted the spinal mass, which appeared to be highly vascular, very dense, and was deforming the spinal cord. We then brought in the intraoperative microscope for further microsurgical dissection and tumor resection.

We utilized D-wave monitoring to provide instant input on the integrity of the lateral corticospinal tract. D-waves, in conjunction with motor evoked potentials, can aid in determining the extent of tumor resection and provide intraoperative warning indicators and alarm criteria for guiding the surgical strategy.

### 4:21 Arachnoid Dissection.

The tumor was found to have a separate plane, and we used this plane to perform a circumferential dissection. For such hypervascular tumors, we generally avoid internal debulking. So, we then coagulated the surface of the tumor to shrink it.

Although this tumor was an intradural intramedullary hemangioblastoma, it exhibited more of an exophytic aspect than a pure intramedullary that required myelotomy for access to the tumor.

In addition, small feeding arteries were coagulated to more effectively control bleeding. The draining vein was preserved until the late stage of the surgery. Sometimes differentiating feeding arteries from draining veins can be challenging, especially when encountering arterialized veins. Compared to draining veins, feeding arteries typically have a brighter pinkish coloration and pulsatile flow. Draining veins are larger and more distended than feeding arteries. The feeding arteries were coagulated.

Here, we then cut the dentate ligament tethered by the tumor. We then found that these actions did not provide a sufficiently substantial reduction of the tumor volume, and therefore we then proceeded with internal debulking of the mass after taking enough arterial feeders.

### 6:08 Tumor Sampling.

We then began central debulking using scissors and tumor forceps with piecemeal resection and with the frequent use of an ultrasonic aspirator. We found that the internal aspect of the mass was highly vascular, requiring a piecemeal resection with frequent hemostasis with the bipolar. Arteries attached to the tumor capsule were then released using microsurgical techniques, where we coagulated only the arterial feeders of the tumor.

Multiple pieces of this mass were sent for permanent pathological analysis. After initial debulking was complete, we continued circumferential dissection. With alternating internal debulking, hemostasis, and piecemeal tumor removal, we were able to shrink the tumor significantly.

### 6:59 Tumor Resection.

Here we found the gliotic plane and we then proceeded to perform a circumferential dissection and piecemeal tumor removal. We identified the feeding arteries, which we then carefully bipolar coagulated. The draining veins were also identified and cautiously bipolar coagulated. We continued circumferential dissection.

The draining veins were also identified and cautiously bipolar coagulated. We then found that the mass was also tethered by dentate ligaments, which we cut and removed. After many hours of a complex microsurgical dissection, we obtained the gross-total resection of the vascular tumor.

### 8:00 Dura, Fascia, and Skin Closure.

We then proceeded with hemostasis of the resection cavity. Once we had achieved hemostasis, we then proceeded to close the dura and other layers in standard fashion.

### 8:09 Postoperative Imaging.

The surgery was uneventful. There was no changes in neurophysiological monitoring. The postoperative period was uncomplicated, and the postoperative MRI of the cervical spine revealed complete resection.

### 8:21 Postoperative Course.

The patient was extubated on the first postoperative day and transferred to general care on the seventh with improvements to her weakness. Her pathology was determined to be a hemangioblastoma of WHO grade 1. Her initial postoperative evaluation demonstrated nearly full strength in her lower limbs, left lower extremity +4/5 and right lower extremity 4/5, along with considerable improvement in her upper limbs, right upper extremity 3/5 and left upper extremity 4/5. After 3 months, her left upper and lower extremities were 5/5. The right upper and lower limbs were both robust. She can now ambulate without a cane and is even able to run to a limited extent and do so independently.

### 9:05 Conclusion.

In conclusion, in the first trimester, therapeutic abortion is recommended for patients with hemangioblastoma who are suspected of having von Hippel–Lindau disease or who have progressive neurological deficits despite receiving symptomatic therapy.^5,6,8^

During the second and third trimesters, patients respond favorably to symptomatic treatment. Corticoids can be used to induce prenatal maturation, but long-term use should be avoided due to the risk of fetal adrenal suppression and neonatal hypoadrenalism. After meeting obstetric criteria, an epidural C-section should be done. Nonetheless, if tumor surgery is required, as in this instance, safe, urgent tumor removal is achievable with close monitoring of the mother and baby. In these conditions, adequate preoperative planning, intraoperative monitoring, and attentive postoperative care are crucial.^5,6,8^

Of note, the decision to conduct instrumented fusion will rely on several variables.^9^ Due to the patient’s complex clinical presentation, the patient’s normal cervical lordosis, the reduction of excessive blood loss, the shortened operation time, and the preservation of the facet joints during the operation, we prefer to constantly monitor the patient for possible spinal instability rather than perform a second spinal surgery.

## Disclosures

The authors report no conflict of interest concerning the materials or methods used in this study or the findings specified in this publication.

## Author Contributions

Primary surgeon: Baskaya. Editing and drafting the video and abstract: Erginoglu, Keles, Ataoglu. Critically revising the work: Baskaya, Erginoglu, Keles, Ataoglu. Reviewed submitted version of the work: all authors. Approved the final version of the work on behalf of all authors: Baskaya. Supervision: Baskaya.

## Supplemental Information

### Patient Informed Consent

The necessary patient informed consent was obtained in this study.
